# Anti-melanoma Differentiation-Associated Gene 5 (Anti-MDA5) Dermatomyositis: A Case Presentation

**DOI:** 10.7759/cureus.23102

**Published:** 2022-03-12

**Authors:** Tyler Ruppel, Jessica Forbes Kaprive, Milad Heydari-Kamjani, Marc M Kesselman

**Affiliations:** 1 Medicine, Nova Southeastern University Dr. Kiran C. Patel College of Osteopathic Medicine, Fort Lauderdale, USA; 2 Dermatology, HCA Lewisgale Montgomery Hospital, Blacksburg, USA; 3 Internal Medicine, Cleveland Clinic Florida, Fort Lauderdale, USA; 4 Rheumatology, Nova Southeastern University Dr. Kiran C. Patel College of Osteopathic Medicine, Fort Lauderdale, USA

**Keywords:** cutaneous manifestations of systemic disease, amyopathic dermatomyositis, progressive interstitial lung disease, inflammatory myositis, anti-mda5 amyopathic dermatomyositis

## Abstract

We present a case of anti-melanoma differentiation-associated gene 5 (Anti-MDA5) dermatomyositis (DM) in a 30-year-old female. Anti-MDA5 dermatomyositis, previously termed clinically amyopathic dermatomyositis, was first recognized in 2005. Most cases present with varying combinations of cutaneous and oral ulcerations, palmar papules, respiratory symptoms, and minor muscle involvement (most commonly in the shoulders, upper arms, hips, thighs, and neck). This subtype of disease is most notable for its association with an increased risk of rapidly progressive interstitial lung disease. Our patient presented initially with only complaints of cutaneous ulcerations on the dorsal aspect of her hands. Following several months of no true diagnosis, she developed muscle weakness and joint pain. This led to retrieval of a punch biopsy which suggested anti-MDA5 DM at the top of the differential diagnoses. Immunoprecipitation revealed the presence of melanoma differentiation-associated gene 5 (MDA5) antibodies, confirming the diagnosis of anti-MDA5 dermatomyositis. This case demonstrates the importance of pinpointing the diagnosis of this rare disease subtype in a timely manner to prevent a fatal course, and we hope to inform dermatologists, rheumatologists, pulmonologists, and internists alike of the uncommon presentation of anti-MDA5 in an unsuspected, young patient.

## Introduction

Anti-melanoma differentiation-associated gene 5 (anti-MDA5) dermatomyositis (DM) has emerged as a unique subtype of classic dermatomyositis first described by Sato et al. in 2005 with distinct mucocutaneous and systemic manifestations [1]. This disease branched from previously termed 'clinically amyopathic dermatomyositis or CADM,' or idiopathic inflammatory myositis, and carries a significant risk for a fatal course if gone unrecognized. The presence of melanoma differentiation-associated gene 5 (MDA5) antibodies in adult patients with DM has been estimated to range anywhere from 7% to 25% [2]. Distinct features of this subtype of disease include mucocutaneous ulcerations, palmar papules, alopecia, panniculitis, arthritis, and most notably, interstitial lung disease [3]. Specifically, the increased risk for interstitial lung disease is associated with a rapidly progressive and fatal course. With early recognition and intervention, however, it is likely that progression to fatal pulmonary sequelae of fibrosis-mediated interstitial lung damage, including diffuse alveolar damage and interstitial fibrosis, may be avoided. We present here a unique case of anti-MDA5 DM in a young woman with an initial, sole presentation of cutaneous ulceration that had gone unrecognized for several months.

## Case presentation

**Figure 1:**
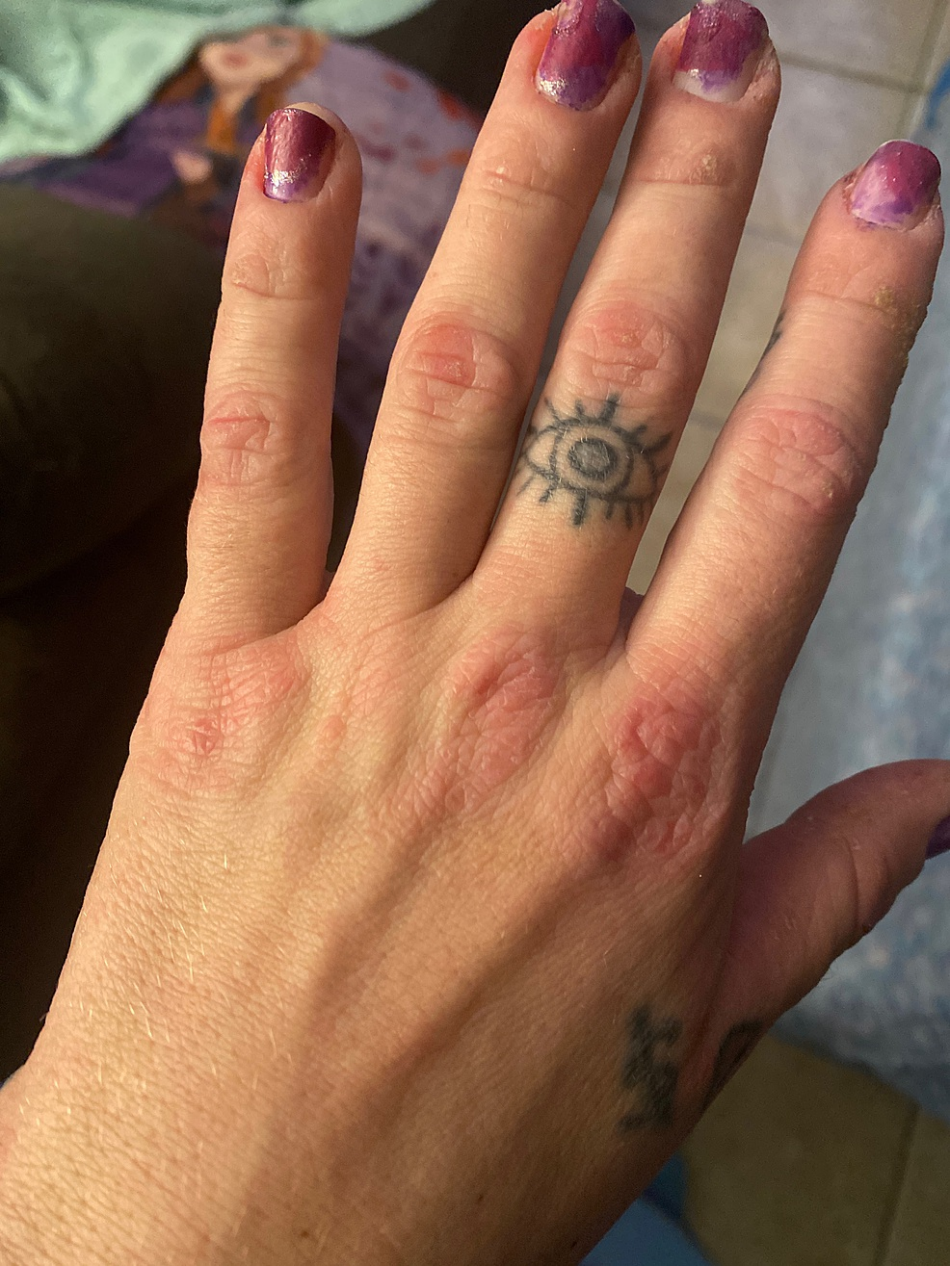
Grotton papules seen on the metacarpophalangeal and interphalangeal joints

**Figure 2:**
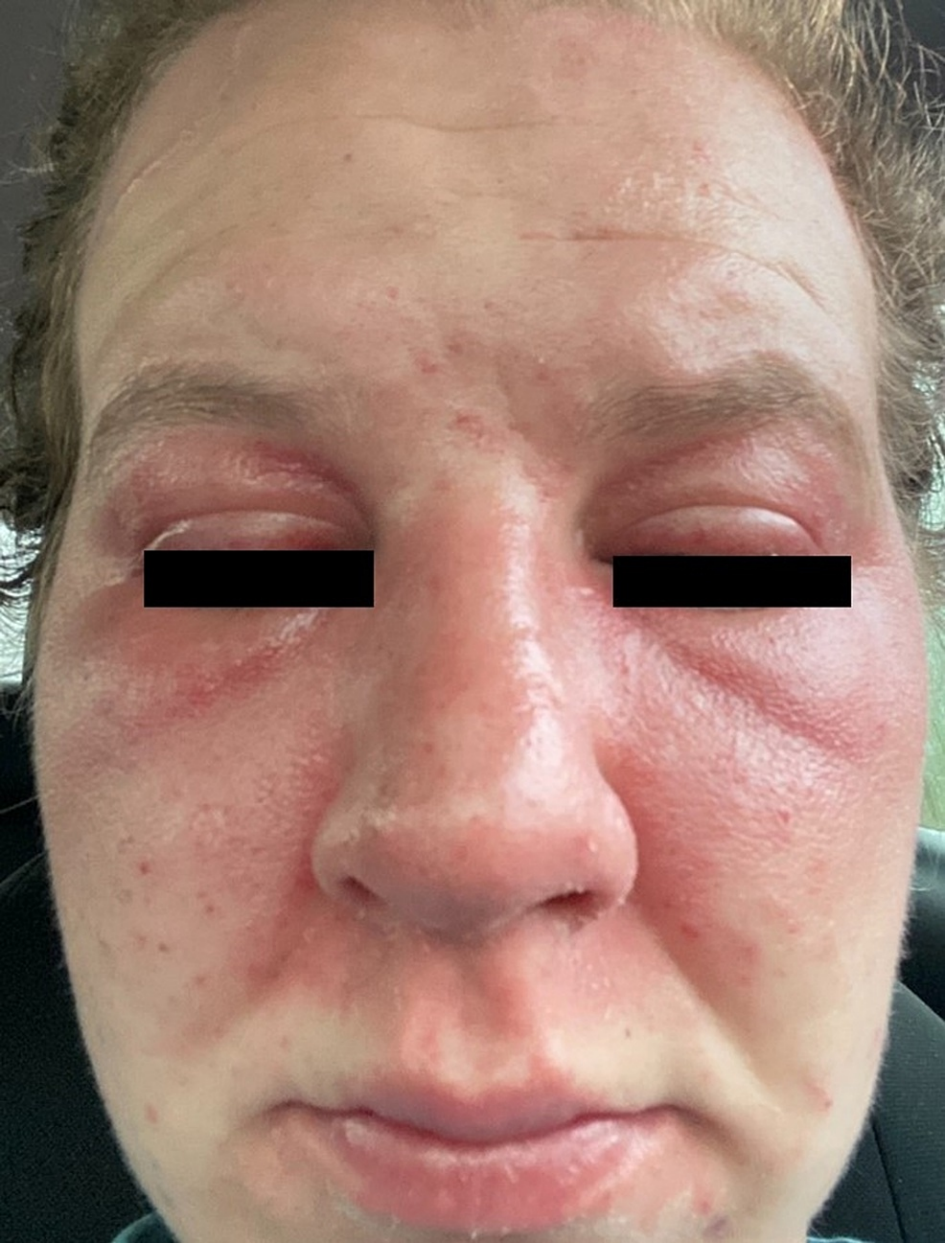
Characteristic heliotrope rash is seen here as a violaceous eruption around upper eyelids and face without sparing the nasal bridge or nasolabial folds. Note, there is sparing of the inter-brow folds unlike in seborrheic keratosis.

**Figure 3:**
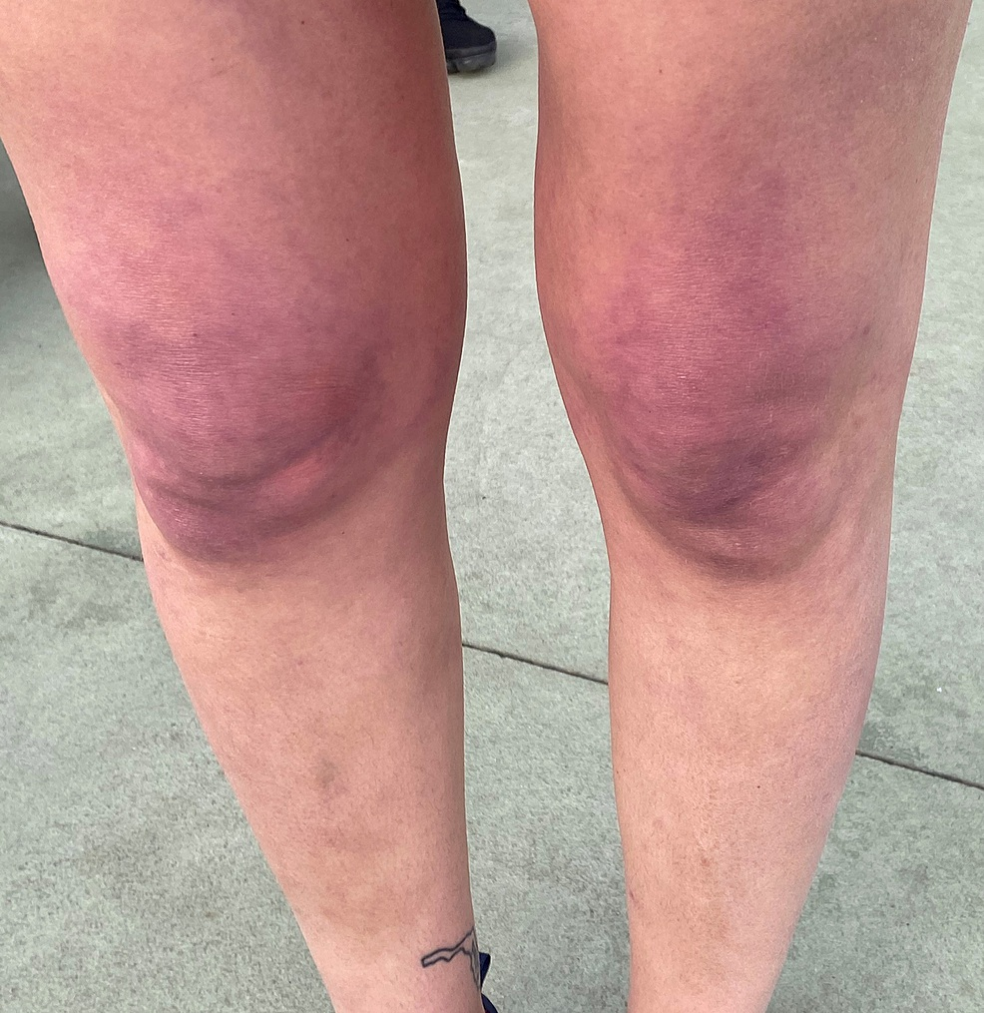
Net-like purple lesions with distinct borders suggestive of livedo reticularis

A 30-year-old female with a past medical history of anemia presented to the rheumatology clinic with joint pain and numerous sores throughout her body as well as brown discolorations on the dorsal aspect of both her hands. She first developed these cutaneous lesions several months before her visit, when she consulted a dermatologist who was unsure of her diagnosis. Months later and upon presentation to us, this patient exhibited associated symptoms of diffuse joint pain and generalized weakness. Physical examination of both her hands revealed papular erythematous lesions on the dorsal aspect of the metacarpophalangeal joints characteristic of Gottron’s papules (Figure 1). Further examination of the patient’s face revealed a violaceous erythema characteristic of a heliotrope rash (Figure 2) and bilateral lower extremities revealed net-like purple lesions with distinct borders suggestive of livedo reticularis (Figure 3). The musculoskeletal examination was significant for proximal muscle weakness with a standard grade of +4/5 and similarily in bilateral shoulders and pelvic girdle. There was also evidence of functional muscle weakness with a simple sit-to-stand test. Radiographic series of the chest and hands were negative for any pathological findings. Punch biopsy of the left dorsal hand was suggestive of DM.

Serological analysis was positive for antinuclear antibodies (ANA) +1:160, human leukocyte antigen B27 (HLA-B27), anti-MDA5 antibody, and suppressed thyroid-stimulating hormone (TSH) levels of 0.22 with free T4 level 1.1 (range: 0.8-1.8). In addition, other notable labs that were checked with negative or within normal were extractable nuclear antigen (ENA) antibody panel negative including Ro (SS-A), La (SS-B), anti-Smith (Sm), ribonucleoprotein (RNP), rheumatoid factor (RF) negative, anti-cyclic citrullinated antibody (CCP) negative, anti-phospholipid antibody, thyroid peroxidase antibody (TPO) negative, antineutrophil cytoplasmic antibodies (ANCA) negative, angiotensin-converting enzyme (ACE) absent in blood, and Lyme serology negative. Serum aldolase was 6.4 U/L (range: 1.0-7.5 U/L), creatine kinase 40.0 U/L (range: 22-198 U/L). Quantiferon labs resulted as indeterminant. Vitamin D levels resulted as 27 ng/ml (normal range: 20-40 ng/ml). 

It is also helpful to note for the investigator that although clinical and serologic testing may be diagnostic for DM, other patients may have equivocal skin findings suggestive of the disease process without muscle involvement. In these cases, a skin biopsy may be beneficial for diagnostic testing. Although histologic comparison of DM is indistinguishable from that of lupus erythematosus (LE), this can aid in distinguishing DM from other disease mimics such as atopic dermatitis, psoriasis, and seborrheic dermatitis. For our patient, all other laboratories including complete blood count (CBC), comprehensive metabolic panel (CMP), RF, CCP, MI-2, Jo-1, signal recognition particle (SRP), TIF-1-gamma, cryoglobulins, erythrocyte sedimentation rate (ESR), and c-reactive protein (CRP) were all within normal limits. The patient was initially started on a course of prednisone and was slowly transitioned to a steroid-sparing therapy, methotrexate.

## Discussion

Anti-melanoma differentiation-associated gene 5 dermatomyositis is a unique subtype of a disease that can differ considerably from typical dermatomyositis leading to an untimely diagnosis and potentially harmful outcomes. The distinction of MDA-5 DM is revealed by the presence of antibodies targeted against MDA5, also known as interferon-induced helicase C domain-containing protein 1 of the innate immune system. The unique clinical features of anti-MDA5 DM include distinct mucocutaneous ulcerations including oral ulcers in up to 82% of cases, as well as palmar papules, calcinosis, panniculitis, arthritis, mechanic's hands, non-scarring alopecia, and most severely, rapidly progressive interstitial lung disease (RP-ILD) [3]. It is known that in patients with MDA5 antibodies, the presence of cutaneous ulcerations is the strongest predictor of the development of RP-ILD [3]. These ulcerations are typically deep, punched out, hyperkeratotic crusts found on the digital pulp, nail folds, elbows, and knees [3]. Moreover, the degree of muscle involvement is under much debate. While the first reports of anti-MDA5 antibodies were described in more than 80% of Japanese patients without muscle involvement, recent studies show that most anti-MDA5 cohorts from the USA demonstrated classic DM with muscle involvement, although with a retained CADM association [1,2].

Diagnosis

A high index of suspicion for anti-MDA5 DM is clinically necessary for patients presenting with unique combinations of the following symptoms: middle-aged female, amyotrophic DM, skin rash, arthritis and/or respiratory symptoms [2]. The official diagnosis of anti-MDA5 DM requires the detection of MDA5 antibodies via immunoprecipitation, the gold standard, or other methods such as immunoassay or enzyme-linked immunoassay (ELISA) [3]. Rapid diagnosis of anti-MDA5 DM is critical in cases of patients presenting with RP-ILD, due to increased risk of mortality. This diagnosis may prove challenging since patients with RP-ILD are likely to present to pulmonologists or intensivists who may not be entirely aware of this rare disease [4]. Thus, careful consideration must be taken of the entire clinical picture, including skin rash or ulceration, joint and muscle involvement, and lung disease.

Lab abnormalities

Additional lab evaluation may help provide prognostic and clinical clues of anti-MDA5 DM, especially in instances when antibody testing is unavailable or delayed. Many patients demonstrate elevated ferritin levels, which appear to be associated with increased disease activity, specifically interstitial lung disease [3]. Experts recommend that patients with elevated ferritin thus receive more aggressive treatment. Levels of ferritin and titers of anti-MDA5 antibodies positively correlate to disease severity and help predict disease prognosis, as well as monitor treatment response. It is also noted that up to 60% of patients have Ro-52 antibodies which also suggest an increased risk for interstitial lung disease as well as cutaneous vasculopathy [2]. Additionally, muscle enzymes including aldolase and creatine kinase may still be elevated [5]. Other lab abnormalities that can be seen include lymphopenia, elevated erythrocyte sedimentation rate, and elevated levels of interleukin-18 in peripheral blood [3].

Management

A new diagnosis of anti-MDA5 positive dermatomyositis warrants a screening for pulmonary disease given the increased risk of RP-ILD. Initial screening includes pulmonary function tests (PFTs) with diffusion capacity for carbon monoxide (DLCO) [2]. If abnormalities are detected, high-resolution computed tomography (CT) of the chest is considered the gold standard [3]. Moreover, while the association between DM and malignancy is well known, most cases of anti-MDA5 DM report a reduced risk for malignancy. However, rare cases of malignancy in anti-MDA5 DM have been reported and thus all dermatomyositis patients, regardless of MDA5 antibody status, should be screened for malignancy [3]. Newly diagnosed dermatomyositis patients should undergo screening for lung, gastrointestinal, reproductive organ, and breast malignancies [6].

Treatment for anti-MDA5 DM consists of using a combination of immunomodulators and immunosuppressants. The initial corticosteroid-sparing agent used in patients with anti-MDA5 DM is mycophenolate mofetil, as it has shown to be effective in previously published case reports [1]. Its mechanism of action in reducing the expression of profibrotic cytokines targets the progression of fibrosis-mediated lung damage seen in interstitial lung disease [7]. Since cutaneous ulceration may be refractory in the treatment of patients with anti-MDA5 DM, intravenous immunoglobulin can be added with mycophenolate mofetil [3]. In progressive pulmonary cases, additional immunosuppressive agents such as rituximab or cyclophosphamide may be advantageous. Refractory ulcerations affected by this vasculopathic disease may be alleviated by therapies that improve circulation such as the vasodilators aspirin, pentoxifylline, nifedipine, sildenafil, and botulinum toxin [3]. Given the variable outcomes of anti-MDA5 DM affecting multifarious organ systems, a multidisciplinary approach is necessary and may warrant expert consultation in pulmonology, rheumatology, dermatology, and wound care.

## Conclusions

We report a 30-year-old female who initially presented with bilateral hand ulcerations and no muscle involvement, unusual for the typical presentation of anti-MDA5 DM. With the potential for a fatal prognosis, anti-MDA5 DM is an immune-mediated, vasculopathic, and ulcerative cutaneous disease with an accompanying increased risk for fibrosis-mediated systemic effects. Although rare, a clinician must recognize early features of the disease process to appropriately screen for life-threatening complications. Since there can be a broad spectrum of clinical diseases linked with MDA5 antibodies and rheumatologic overlap among other disease processes, it is imperative to keep anti-MDA5 dermatomyositis on the differential.
